# Predictors of early progression to severe sepsis or shock among emergency department patients with nonsevere sepsis

**DOI:** 10.1186/s12245-016-0106-7

**Published:** 2016-02-24

**Authors:** Andre L. Holder, Namita Gupta, Elizabeth Lulaj, Miriam Furgiuele, Idaly Hidalgo, Michael P. Jones, Tiphany Jolly, Paul Gennis, Adrienne Birnbaum

**Affiliations:** Department of Medicine, Emory University School of Medicine, 1648 Pierce Dr NE, Atlanta, GA 30307 USA; Beacon Health System, Elkhart General Hospital, 600 East Blvd, Elkhart, IN 46514 USA; Department of Radiology, Emory University School of Medicine, 1648 Pierce Dr NE, Atlanta, GA 30307 USA; Department of Emergency Medicine, Albert Einstein College of Medicine, Montefiore Medical Center, 111 East 210th Street, Rosenthal Red Zone, Room 2nd Floor, Bronx, NY 10467 USA; Department of Emergency Medicine, Kingston Hospital, 396 Broadway, Kingston, NY 12401 USA; Department of Emergency Medicine, Albert Einstein College of Medicine, Jacobi Medical Center, 1400 Pelham Parkway South, 1B-25, Bronx, NY 10461 USA; Department of Emergency Medicine, Saint Francis Hospital and Medical Center, 114 Woodland Street, Hartford, CT 06105 USA; Division of Pulmonary, Allergy, Critical Care and Sleep Medicine, Emory University School of Medicine, Grady Memorial Hospital, 80 Jesse Hill Jr. Drive SE, Rm 2D012, Atlanta, GA 30303 USA

**Keywords:** Disease progression, Nonsevere sepsis, Organ dysfunction, Predictors, Sepsis progression

## Abstract

**Background:**

Progression from nonsevere sepsis—i.e., sepsis without organ failure or shock—to severe sepsis or shock among emergency department (ED) patients has been associated with significant mortality. Early recognition in the ED of those who progress to severe sepsis or shock during their hospital course may improve patient outcomes. We sought to identify clinical, demographic, and laboratory parameters that predict progression to severe sepsis, septic shock, or death within 96 h of ED triage among patients with initial presentation of nonsevere sepsis.

**Methods:**

This is a retrospective cohort of patients presenting to a single urban academic ED from November 2008 to October 2010. Patients aged 18 years or older who met criteria for sepsis and had a lactate level measured in the ED were included. Patients were excluded if they had any combination of the following: a systolic blood pressure <90 mmHg upon triage, an initial whole blood lactate level ≥4 mmol/L, or one or more of a set of predefined signs of organ dysfunction upon initial assessment. Disease progression was defined as the development of any combination of the aforementioned conditions, initiation of vasopressors, or death within 96 h of ED presentation. Data on predefined potential predictors of disease progression and outcome measures of disease progression were collected by a query of the electronic medical record and via chart review. Logistic regression was used to assess associations of potential predictor variables with a composite outcome measure of sepsis progression to organ failure, hypotension, or death.

**Results:**

In this cohort of 582 ED patients with nonsevere sepsis, 108 (18.6 %) experienced disease progression. Initial serum albumin <3.5 mg/dL (OR 4.82; 95 % CI 2.40–9.69; *p* < 0.01) and a diastolic blood pressure <52 mmHg at ED triage (OR 4.59; 95 % CI 1.57–13.39; *p* < 0.01) were independently associated with disease progression to severe sepsis or shock within 96 h of ED presentation. There were no deaths within 96 h of ED presentation.

**Conclusions:**

In our patient cohort, serum albumin <3.5 g/dL and an ED triage diastolic blood pressure <52 mmHg independently predict early progression to severe sepsis or shock among ED patients with presumed sepsis.

## Background

Recent literature suggests that patients with sepsis who develop organ dysfunction represent approximately 25 % of those who initially present to the emergency department (ED) with sepsis [1, 2]. Mortality estimates for severe sepsis and septic shock often include this group of patients [3]. However, the mortality may be higher in those whose condition progresses in the hospital compared with those who present to the ED with organ dysfunction. One study demonstrated a 20 % higher absolute hospital mortality among septic patients who developed shock late in their hospital course compared to those who had shock early [4]. Timely identification of patients with nonsevere sepsis—i.e., those without organ dysfunction or shock—who later develop severe sepsis may impact patient morbidity and mortality.

While attention has been placed on early, appropriate treatment of severe sepsis and septic shock in the ED [5–10], no treatment strategies have been devised targeting patients with nonsevere sepsis. One reason is that it is difficult to identify patients who are at risk for progression to severe sepsis or septic shock. Patients with sepsis who already have signs of organ dysfunction are at risk for further disease progression [1, 2], but little is known about those without any initial evidence of organ damage upon ED presentation. Early identification of patients with nonsevere sepsis at highest risk for progression is a critical first step in preventing sepsis progression. The health-care resources deployed at a later stage in the disease process could be more appropriately directed toward earlier aggressive therapeutic interventions in the ED. Early prediction of disease progression in sepsis could also be used for timely triage to a higher level of care upon admission.

Prognostication rules exist to predict mortality among septic patients in the ED [3, 11, 12]; but currently, there are no such tools to predict the development of organ dysfunction or shock in the subset of patients with nonsevere sepsis who present to the ED. The first step in developing a prediction tool is to identify clinical predictors of disease progression in patients with nonsevere sepsis. While a few studies have tried to determine the predictors of progression in the ED, some have limited the patient population to those who have proven sepsis. This approach may be more diagnostically accurate; but often, clinicians in the ED act on a suspicion of infection since proof of infection may only develop over time in the inpatient setting. If the goal is to derive parameters that could be used as an ED screening tool, then limiting the study population to those with proven infection would not facilitate real-time ED-based prediction.

We hypothesized that there are clinical, demographic, and laboratory parameters that independently predict early sepsis progression. The goal of this study is to identify specific parameters that are associated with a composite outcome of progression to severe sepsis, shock, or death within 96 h of ED triage among ED patients who initially present with nonsevere sepsis.

## Methods

### Design

This was a retrospective single-center cohort study of adult ED patients with suspected sepsis. A computerized decision support system in the electronic medical record was used to screen for eligible patients from November 2008 to October 2010. The institutional review board of the study site approved the study with a waiver of informed consent.

### Setting and population

Patient records were reviewed for all patients presenting to the ED at the Jacobi Medical Center, an academic teaching hospital in the Bronx, NY, with an annual ED census of 75,000 patients. Patients were included in the study if they (1) were 18 years of age or older, (2) had sepsis (two out of four SIRS criteria and suspicion of infection based on an accepted consensus definition [13]), and (3) had an initial lactate level <4 mmol/L measured within 6 h of ED presentation. Suspicion of infection was confirmed by a clinician query in the electronic medical record (below).

Eligible patients were first identified by a query of the QuadraMed health information system (QuadraMed Corporation; Weston, VA) as performed by the Jacobi Medical Center information technology (IT) department using a preexisting electronic decision support system. The support system automatically combined triage vital signs with the white blood cell count then alerted the clinician that lactate testing was recommended for patients with suspected infection who meet two or more SIRS criteria. Clinicians were electronically queried about the presence or absence of infection upon completion of the ED electronic medical record once a clinical disposition was recorded.

Patients were excluded from the study if they (1) had a systolic blood pressure less than 90 mmHg upon ED triage and (2) exhibited any signs of organ dysfunction upon initial evaluation. The organ systems assessed were respiratory, renal, coagulation, hematologic, hepatic. We used a slightly modified form of standard definitions provided in a prior paper (Table 1) [14]. Patients were also excluded if they (3) were discharged from the ED, (4) were made DNR prior to or at any time during the hospital visit of interest, or (5) left against medical advice. If patients had more than one encounter that met the inclusion criteria, only the first visit was included in the sample and any subsequent visits were excluded. Five residents (NG, MF, IH, MJ, TJ), one attending emergency physician (AH), and one physician volunteer (EL) evaluated the patient charts for inclusion in the study using a standardized data collection instrument (DCI).

**Table 1:** Modified definitions for organ dysfunction

Organ dysfunction	Original definitions	Modified definitions
Respiratory	paO_2_/FiO_2_ < 300	Intubation
Renal	Creatinine increase >0.5 mg/dL or urine output <0.5 mL/kg/h for at least 2 h	Creatinine increase >0.5 mg/dL^a^
Coagulopathy	INR >1.5 or aPTT >60 s	n/a
Hematologic	Platelet count <100,000/uL	n/a
Liver	Total bilirubin >4 mg/dL	n/a

*paO2* arterial partial pressure of oxygen, *FiO2* fractional oxygen percentage, *INR* international normalized ratio, *aPTT* activated partial thromboplastin time

^a^If a patient did not have a prior visit and the initial creatinine >2.0 mg/dL, we assessed if there was a >50 % decrease in creatinine in the first 96 h of hospital care

### Data

Easily accessible variables that are common and standard in most ED practices were used as candidate variables for regression modeling and were retrieved from a variety of sources (Table 2). Basic demographic information such as age and sex, physiologic markers such as diastolic blood pressure, and laboratory data were all retrieved retrospectively from the electronic medical record. Specific variables were chosen based on suspected or proven clinical relevance shown in prior studies [1, 12, 15–22]. The data used for the composite outcomes of organ dysfunction and shock were extracted in a similar manner. Other factors in the existing literature proven to predict mortality among septic ED patients include the following: whether the patient came from a nursing home, whether the patient had a comorbid lung disease, and whether he/she had a vascular access such as a dialysis catheter [1]. This information was retrospectively extracted from the chart. Cutoff values were chosen for all continuous variables to simplify predictors, making them easier to enter into models utilized in a busy clinical setting (Table 2).

**Table 2:** Methods of measurement for potential predictors of sepsis progression

Variable category	Variable	Categories chosen (when applicable)
Demographic predictors	Age (years)	
	Race	
	Sex	
Clinical predictors	Nursing home resident status	
	Suspicion of lower respiratory tract infection	
	Presence of long-term vascular access	
	Triage diastolic blood pressure (mmHg)	<52 mmHg; ≥52 mmHg
Laboratory predictors	Serum bicarbonate (mEq/L)	<20 mEq/L; ≥20 mEq/L
	Serum hemoglobin (g/dL)	<10 g/dL; >10 g/dL
	Serum albumin (g/dL)	<3.5 g/dL; ≥3.5 g/dL
	Serum sodium (mEq/L)	>145 mEq/L; ≤145 mEq/L
	Serum glucose (mg/dL)	<60 or >300 mg/dL; 60–300 mg/dL
Comorbidities	Diabetes mellitus	
	Coronary artery disease	
	Congestive heart failure	
	Cirrhosis	
	Chronic renal disease	
	Chronic obstructive pulmonary disease/asthma	
	HIV/AIDS	
	Alcohol dependence	
	Cancer	
Organ dysfunction	Creatinine (mg/dL)	
	INR	
	Activated partial prothrombin time (aPTT) (s)	
	Platelet count (uL^−1^)	
	Total bilirubin (mg/dL)	
Tissue hypoperfusion	Lactate (mmol/L)	
Shock	Systolic blood pressure (mmHg)	
	Need for vasopressors	

Trained research associates abstracted the data from the medical records of all septic patients with a lactate level sent during the study period. The primary investigator (AH) met with all research associates collectively and individually to provide a standardized orientation on how data should be extracted. The data abstractors were audited weekly. Four reviewers abstracted the data on potential predictor variables from the ED summaries, admission notes, and hospital discharge summaries. Three independent reviewers abstracted information about the presence of the outcome of interest—progression of patients in nonsevere sepsis to severe sepsis or death within 96 h of ED triage. Both groups were blinded to the information collected by the other group. The abstractors used REDCap (Vanderbilt University; Memphis, TN), a secured internet data management computer program, to enter the data on both predictors and outcomes. REDCap utilizes online DCIs and allows automatic entry into a spreadsheet, which obviates the need for manual data entry to a spreadsheet and minimizes transcription error. DCIs were designed for both predictor and outcome variables. Ten percent of patients had all variables collected by a second unblinded abstractor (AH or NG) to confirm reliability of data.

Information on underlying comorbidities was collected by chart review. Three reviewers (AH, NG, EL) searched the electronic medical record in the ED summaries, admission notes, and discharge summaries looking for the following comorbidities: diabetes mellitus (DM), coronary artery disease (CAD), congestive heart failure (CHF), cirrhosis and/or diagnosis of hepatitis B and/or C, chronic kidney disease, chronic obstructive pulmonary disease (COPD), asthma, acquired immune deficiency syndrome (AIDS), alcohol dependence, and cancer. These secondary diagnoses were chosen based on previously reported associations with sepsis mortality [19–21].

All relevant patient information collected via manual chart review of the electronic medical record was assessed for interrater reliability. All variables collected by the DCIs showed at least good agreement (kappa = 0.82 for exclusion DCI; kappa = 0.85 for the “nursing home” variable; kappa = 0.67 for the “clinical suspicion of pneumonia in ED” variable; kappa = 1.00 for the “patient presents with a dialysis catheter” variable; kappa = 0.79 for the “disease progression” variable).

The primary outcome is a composite measure of sepsis progression. Disease (sepsis) progression was defined as the development of one or more of the following within 96 h of ED presentation: (1) organ dysfunction (Table 1); (2) a lactate level ≥4 mmol/L; (3) shock, defined as at least one measurement of systolic blood pressure <90 mmHg, and/or initiation of vasopressor therapy; or (4) death. We chose disease progression up to 96 h from ED presentation to identify sepsis progression that occurs early in the hospital course.

There was a significant number of missing values—either initial or repeat measurements—among many of the outcome variables that comprised the composite outcome. This was handled in two ways. First, it was assumed for the composite primary outcome that patients who only had an initial laboratory value level sent for any given organ system did not go on to develop dysfunction of that organ. Patients would be less likely to have a repeat test ordered if their physicians did not suspect disease progression. Secondly, a sensitivity analysis was performed using only those outcome variables with <5 % missing values. This “reduced composite outcome variable” of disease progression was decided upon post hoc and used only respiratory failure, renal dysfunction, thrombocytopenia, and/or the development of shock. (Though the respiratory failure outcome was missing in >5 % of cases, it was also included since it would be very unlikely that a patient would be intubated without an X-ray to confirm proper placement of the endotracheal tube. If a patient did not have a repeat X-ray within 96 h of their ED presentation, he/she likely did not have respiratory dysfunction).

### Statistical analysis

The relationship between each candidate predictor and the primary composite outcome of sepsis progression was assessed using univariate analyses. All continuous variables were converted to categorical variables in an effort to find clinically meaningful cutoffs. Pearson’s chi-squared or Fisher’s exact tests were used for categorical variables, as appropriate. All variables that were statistically significant at an alpha <0.25 in univariate analysis were included in a logistic regression model. (Age was included in the model regardless of statistical significance in univariate analysis). Wald statistics were used to assess each variable’s statistical significance in the model. An alpha of 0.05 decided whether a variable would be retained in the model. Regression diagnostics were assessed using the Hosmer and Lemeshow goodness-of-fit test. Influential datapoints and covariate patterns were checked using the delta chi-squared and delta deviance influence statistics. Tests for interaction were conducted between predefined groups of variables.

All statistical analysis was conducted with STATA 10.1 (StataCorp LP, College Station, TX, 2009).

## Results

Nine hundred ninety-five patients met the inclusion criteria during the study period from November 2008 to October 2010. After applying the exclusion criteria and subsequently excluding three patients who signed out of the hospital against medical advice, there were 582 eligible patients found in our chart review who had nonsevere sepsis in the emergency department. One hundred eight (18.6 %) of the patients meeting the inclusion criteria went on to develop the composite outcome of severe sepsis or shock within 96 h of ED presentation. (No patients died within this time period). Table 3 demonstrates key clinical characteristics about the patient cohort.

**Table 3:** Characteristics of patients with nonsevere sepsis upon emergency department (ED) presentation

Sample characteristics	*n* = 582
Age (years), mean (SD)	56.1 (17.3)
Sex (women), *n* (%)	276 (47.7)
Race	
Hispanic, *n* (%)	287 (49.3)
Blacks, *n* (%)	149 (25.6)
White, *n* (%)	66 (11.3)
Other, *n* (%)	80 (13.7)
Initial WBC count (mg/dL), median (IQR)	12.3 (6.6)
Pulse at ED triage (bpm), mean (SD)	103.7 (21.5)
Respiratory rate at ED triage (bpm), mean (SD)	18 (2)
Temperature at ED triage (°F), median (IQR)	99.5 (3.4)
Diastolic blood pressure at ED triage (mmHg), mean (SD)	75.7 (14.3)
Serum hemoglobin (g/dL), mean (SD)	12.2 (2.1)
Serum albumin (g/dL), mean (SD)	3.8 (0.6)
Serum bicarbonate (mEq/L)	26.0 (4.8)
Serum sodium (mEq/L), mean (SD)	137.9 (4.6)
Serum glucose (mg/dL), median (IQR)	121 (70)
Nursing home residents, *n* (%)	95 (16.3)
Clinical suspicion for pneumonia in the ED, *n* (%)	158 (27.1)
History of diabetes, *n* (%)	201 (34.5)
History of congestive heart failure, *n* (%)	57 (9.8)
History of coronary artery disease, *n* (%)	68 (11.7)
History of cirrhosis and/or hepatitis B and/or C, *n* (%)	52 (8.9)
History of chronic kidney disease, *n* (%)	62 (10.6)
History of chronic obstructive pulmonary disease or asthma, *n* (%)	124 (21.3)
History of stroke, *n* (%)	58 (10.0)
History of AIDS (CD4 <200), *n* (%)	26 (4.5)
History of cancer, *n* (%)	66 (11.3)
History of alcohol abuse, *n* (%)	32 (5.5)

Nonsevere sepsis defined as patients meeting two out of four SIRS criteria with suspected sepsis, with no evidence of organ dysfunction (severe sepsis) or shock

*WBC* white blood cell, *SD* standard deviation, *IQR* interquartile range, *AIDS* acquired immune deficiency syndrome

Table 4 shows that age, sex, race, hemoglobin, diastolic blood pressure <52 mmHg at ED triage, initial hemoglobin <10 mg/dL, initial albumin <3.5 g/dL, serum bicarbonate <20 mEq/L, initial sodium level >145 mEq/L, initial serum glucose >300 or <60 mg/dL, nursing home residence, clinical suspicion for pneumonia in the ED, presence of a dialysis catheter, and a history of diabetes, congestive heart failure, coronary artery disease, liver disease, chronic kidney disease, obstructive lung disease, stroke, AIDS, cancer and/or alcohol abuse were all included in the model. We found no interactions between variables.

**Table 4:** Predictor variables and their relationship with disease progression among septic patients

Variable	Univariate model			Multivariate model		
	Odds ratio	95 % CI	*p* value	Odds ratio	95 % CI	*p* value
Age			0.18			0.17^a^
1st age quartile (18–44 years)	(Ref)	(Ref)		1.88	1.00–3.54	
2nd age quartile (45–55 years)	1.81	0.98–3.35		1.63	0.87–3.06	
3rd age quartile (56–68 years)	1.75	0.95–3.20		1.17	0.61–2.24	
4th age quartile (69–101 years)	1.28	0.68–2.40				
Diastolic (<52 mmHg)	4.66	1.71–12.71	<0.01	4.59	1.57–13.39	<0.01
Hemoglobin (<10 g/dL)	1.48	0.87–2.51	0.15			
Albumin (<3.5 g/dL)	5.11	2.60–10.0	<0.01	4.82	2.40–9.69	<0.01
Bicarbonate (<20 mEq/L)	1.88	0.90–3.92	0.09			
Serum sodium (>145 mEq/L)	2.03	0.85–4.79	0.11			
Serum glucose (>300 or <60 mg/dL)	0.63	0.27–1.43	0.27			
Nursing home resident	1.51	0.89–2.55	0.12			
Race			0.92			
Hispanics	(Ref)	(Ref)				
Blacks	0.87	0.52–1.46				
Whites	1.06	0.54–2.08				
Other	1.08	0.58–2.01				
Sex (women)	1.19	0.78–1.80	0.42			
Clinical suspicion for pneumonia in the ER	1.37	0.87–2.15	0.17			
Patient has a dialysis catheter	0.73	0.16–3.29	0.68			
History of DM	0.84	0.54–1.32	0.46			
History of CHF	0.93	0.45–1.90	0.84			
History of CAD	1.41	0.77–2.58	0.26			
History of liver disease^b^	1.71	0.89–3.28	0.11			
History of kidney disease^c^	1.46	0.79–2.73	0.23			
History of obstructive lung disease^d^	1.38	0.85–2.24	0.19			
History of CVA	1.79	0.96–3.32	0.06			
History of AIDS	2.44	1.05–5.64	0.04			
History of cancer	1.47	0.80–2.70	0.21			
History of alcohol abuse	1.24	0.52–2.96	0.62			

*CI* confidence interval, *DM* diabetes mellitus, *CHF* congestive heart failure, *CAD* coronary artery disease, *CVA* cerebrovascular accident, *AIDS* acquired immune deficiency syndrome, defined as a CD4 count <200

^a^Included in the model a priori

^b^Liver disease was defined as cirrhosis or a diagnosis of hepatitis B or C

^c^Kidney disease was defined as renal replacement therapy (dialysis) or creatinine >2

^d^Obstructive lung disease was defined as COPD or asthma

Among patients initially presenting to the ED in nonsevere sepsis, an initial serum albumin <3.5 g/dL (OR 4.82; 95 % CI 2.40–9.69) and an ED triage diastolic blood pressure <52 mmHg (OR 4.59; 95 % CI 1.57–13.39) were both independently associated with the development of severe sepsis or shock within 96 h of ED presentation. There were no deaths within the first 96 h of ED presentation in our sample. History of AIDS had an OR 2.23 to predict disease progression when controlling for age, diastolic blood pressure, and serum albumin, but this relationship did not reach statistical significance [*p* = 0.08; not shown].

Table 5 demonstrates that when we used the reduced composite outcome of disease progression as the outcome in our model, only an initial serum albumin <3.5 g/dL was independently associated with progression to severe sepsis or shock among patients presenting to the ED in nonsevere sepsis (OR 5.61; 95 % CI 2.80–11.24). None of the associations analyzed with the primary composite outcome of disease progression that were not statistically significant became statistically significant when we applied the reduced composite outcome of disease progression.

**Table 5:** Predictor variables and their relationship with the reduced composite disease progression outcome among septic patients

Variable	Univariate model			Multivariate model		
	Odds ratio	95 % CI	*p* value	Odds ratio	95 % CI	*p* value
Age			0.32			0.39^a^
1st age quartile (18–44 years)	(Ref)	(Ref)		(Ref)	(Ref)	
2nd age quartile (45–55 years)	1.60	0.85–3.05		1.56	0.81–2.98	
3rd age quartile (56–68 years)	1.61	0.86–3.00		1.43	0.75–2.71	
4th age quartile (69–101 years)	1.14	0.59–2.19		1.01	0.52–1.97	
Diastolic (<52 mmHg)	3.13	1.09–9.00	0.03			
Hemoglobin (<10 g/dL)	1.21	0.68–2.14	0.51			
Albumin (<3.5 g/dL)	5.63	2.83–11.2	<0.01	5.61	2.80–11.24	<0.01
Bicarbonate (<20 mEq/L)	1.81	0.84–3.89	0.13			
Serum sodium (>145 mEq/L)	2.09	0.88–4.94	0.09			
Serum glucose (>300 or <60 mg/dL)	0.64	0.23–1.47	0.30			
Nursing home resident	1.53	0.89–2.62	0.12			
Race			0.58			
Hispanics	(Ref)	(Ref)				
Blacks	0.72	0.41–1.25				
Whites	1.04	0.52–2.10				
Other	1.13	0.60–2.13				
Sex (women)	1.23	0.79–1.89	0.35			
Clinical suspicion for pneumonia in the ER	1.31	0.82–2.09	0.26			
Patient has a dialysis catheter	0.81	0.18–3.73	0.79			
History of DM	0.78	0.49–1.25	0.30			
History of CHF	0.87	0.41–1.84	0.71			
History of CAD	1.22	0.65–2.30	0.54			
History of liver disease^b^	1.89	0.95–3.73	0.07			
History of kidney disease^c^	1.14	0.58–2.24	0.70			
History of obstructive lung disease^d^	1.31	0.79–2.18	0.29			
History of CVA	1.81	0.96–3.42	0.07			
History of AIDS	2.68	1.15–6.26	0.02			
History of cancer	1.38	0.74–2.57	0.31			
History of alcohol abuse	1.40	0.58–3.35	0.45			

The reduced composite outcome includes respiratory failure, renal dysfunction, thrombocytopenia, or development of shock (see text for details)

*CI* confidence interval, *DM* diabetes mellitus, *CHF* congestive heart failure, *CAD* coronary artery disease, *CVA* cerebrovascular accident, *AIDS* acquired immune deficiency syndrome, defined as a CD4 count <200

^a^Included in the model a priori

^b^Liver disease was defined as cirrhosis or a diagnosis of hepatitis B or C

^c^Kidney disease was defined as renal replacement therapy (dialysis) or creatinine >2

^d^Obstructive lung disease was defined as COPD or asthma

While there were no deaths within 96 h of presentation to the ED, our sample had five (5) deaths throughout the hospital course; 3 (0.63 %) of them did not have disease progression within 96 h of ED presentation, while the remaining 2 (1.85 %) did.

## Discussion

In the present study, almost one in five patients presenting to the ED with sepsis experienced disease progression. This incidence is in keeping with prior reports in the literature, which range from 12 to 26 % [1, 2, 23, 24]. Severe sepsis and septic shock carry a high mortality burden [25], which warrants a means by which clinicians can predict those at highest risk for disease progression. Our study sought to find factors associated with development of organ failure, shock, or death among ED patients presenting with nonsevere sepsis. We found that an admission albumin less than 3.5 g/dL and an ED triage diastolic blood pressure less than 52 mmHg were independently associated with the development of organ failure or shock in sepsis within 96 h of ED presentation.

The two associations we found are biologically plausible. Serum albumin is commonly considered a marker of nutritional status, but the reasons for hypoalbuminemia among hospitalized patients are complex. In a metabolically stressed state, decreased enteral protein intake and increased intravascular catabolism are partly responsible, but the two primary reasons for low albumin levels in sepsis are (1) increased transcapillary loss and (2) impaired hepatic production. Under normal circumstances, the transcapillary escape rate (TER) of albumin—i.e., the amount of intravascular albumin that leaves the bloodstream in the capillary beds—is about 5 % per hour. That amount increases by about twofold in patients with sepsis [26], and up to threefold among those with septic shock [27]. This suggests that the degree of albumin leak—and thus hypoalbuminemia—is correlated with sepsis progression. Cytokines such as interleukin-2, interferon-alpha, and interleukin-6 are hypothesized to be the main mediators for increased capillary leak in acute inflammatory states like sepsis. One study demonstrated a reliable increase in TER after exogenous interleukin-2 injection [28].

Cytokines also directly suppress albumin production by the liver. Albumin has anti-inflammatory effects, including decreased platelet aggregation [29], so its production is likely downregulated to bolster the body’s inflammatory response. However, the effects of cytokines on hepatic production take hours to days [30] and probably contributes more to hypoalbuminemia in states of persistent organ failure. Whatever the main mechanistic cause, the effects of inflammation on serum albumin levels have important consequences beyond sepsis progression; hypoalbuminemia is associated with higher mortality among patients with severe sepsis and septic shock [31].

A diastolic blood pressure of 52 mmHg corresponds with a mean arterial pressure of 65 mmHg and a systolic blood pressure of 90 mmHg. The latter two values are defined as clinical hypotension. Though a patient may not be hypotensive by mean arterial pressure or by systolic blood pressure in the ED, a low diastolic blood pressure upon triage may be an early, subtle sign that a septic patient may go on to have disease progression. Diastolic blood pressure is one marker of vascular tone. One study demonstrated that levels of endogenous inflammatory mediators such as nitrous oxide and TNF-alpha are associated with progression to severe sepsis, shock, or death [32]. These cytokines and autocrine hormones are known vasodilators. Septic patients who have normal mean and systolic pressures with low diastolic pressures are in a compensated vasodilated state, a precursor to more overt cardiovascular collapse.

The shared pathophysiology of hypoalbuminemia and low diastolic blood pressure and what they represent in sepsis add credibility to our findings. The infectious pathogen and damaged host tissue create a cytokine and chemokine “storm” in the early phase of sepsis that activates the innate and adaptive host responses. Hypoalbuminemia and low diastolic blood pressure are markers of two microcirculatory abnormalities—loss of endothelial barrier function and low capillary perfusion pressure, respectively—caused by extensive cytokine-derived endothelial damage. Those microcirculatory derangements are major contributors to organ failure in sepsis [33].

Early identification and prevention of organ dysfunction or hemodynamic compromise highlights a very important principle in the treatment of patients with sepsis. The early goal-directed therapy trial by Rivers et al. [5] showed a clinically and statistically significant mortality benefit of protocol-based care among patients with early severe sepsis or septic shock. Recent evidence has suggested that early recognition, early antibiotics, and early fluid resuscitation are likely the key ingredients in early goal-directed therapy [34–36]. Identifying patients who are at risk for subsequent disease progression to severe sepsis or septic shock could preclude the need for instituting aggressive care later in the hospital course if it begins in the ED to prevent organ dysfunction and cardiovascular collapse.

Our study adds useful information to the pool of available studies that seek to identify factors associated with disease progression in sepsis [1, 2, 16, 23, 24, 37–40]. First, this is an ED-based study of a large-sized patient cohort. The goal was to focus on readily accessible information in the ED that can be utilized by emergency physicians either as a tool to increase the triage level of those identified as being higher risk or to more aggressively treat patients with nonsevere sepsis prior to ED disposition. There are a few studies that attempt to identify sepsis patients at risk for clinical decompensation in the inpatient setting [16, 39], but these patients are likely to represent a different population with different risk factors for disease progression. Second, we specifically sought out patients with a suspicion for infection, unlike other studies that sought patients with confirmed infection [1]. Glickman et al. [1] only enrolled those who had confirmed infection by either microbiology or other objective diagnostic testing. This information is often unavailable at the time of ED presentation, potentially limiting its applicability in real time to the undifferentiated ED population of patients with suspected sepsis. Though many other studies have used clinician suspicion of infection as an entry criterion, many use billing data [39] or complicated chart review algorithms [23] to imply that there was clinical suspicion of sepsis during the ED encounter. To our knowledge, our study is the first to capture clinician judgment in real time through clinician documentation in the electronic medical record. Third, we specifically sought to enroll only those patients who had no signs of end organ damage at enrollment. This is a group of patients often overlooked in existing studies about disease progression. Capp et al. [23] found that female gender, transient hypotension, bandemia, past history of coronary artery disease, and lactate greater than 4 mmol/L were all associated with development of septic shock. Patients with a lactate greater than 4 mmol/L were excluded from our study since this was among the outcomes included in our composite disease progression outcome of interest. Arnold et al. [2] identified risk factors for organ dysfunction among patients classified as “preshock,” defined as patients with a lactate of 2–4 mmol/L without hypotension or the need for mechanical ventilation or vasopressor support. However, patients had a median sequential organ failure assessment (SOFA) score of 2 upon presentation to the ED; half of their cohort started with a SOFA score above 2, indicating either significant single organ failure or early multiorgan dysfunction. Any associated variables found in this study may not be applicable to the patients of interest in our study. Last, our outcome of interest is a composite outcome that included definitions of severe sepsis based on widely accepted international guidelines (Table 1) [14]. This allowed for an objective, reproducible outcome. Arnold et al. [2] used a composite outcome that defined worsened organ dysfunction as an increase in SOFA score of greater than 1; it is hard to identify which organ system is affected or to quantify that progression with such a measure, making it hard to use the results in clinical practice. The outcome of interest in a paper by Kennedy et al. [37] was ICU transfer. While they retrospectively identified only those transfers that were not due to clinical decompensation, clinicians may have different thresholds for transferring patients to the ICU, potentially limiting clinical applicability of any models derived with this outcome.

Though the factors we found to be associated with sepsis progression are plausible, several other variables failed to exhibit similar relationships. Glickman et al. found an association with age and disease progression [1], though this has not been replicated in other studies [23, 24, 37, 39]. Conflicting conclusions could be due to a difference in disease burden between studies. The median APACHE II score among the cohort admitted to a hospital ward studied by Whittaker et al. [39] was 13, whereas the patient cohort in the study by Glickman et al. [1] had a median APACHE II score of 9. Our study found no independent association between age and disease progression in sepsis, though we decided a priori to retain age in our multivariate model. Coming from a nursing home was also not independently associated with disease progression in sepsis (Tables 4, 5, and 6). This is comparable to the finding by Song et al. [24] who found no statistically significant difference in the percentage of patients from a nursing home with disease progression compared with those from a nursing home without progression. The prevalence of nursing home residents in their cohort was only 3 %, compared to 16.3 % in our cohort. Perhaps, nursing home residents were treated with more aggressive antibiotics and more frequent fluid boluses than their non-nursing home counterparts, thus eliminating any higher likelihood of disease progression that would otherwise exist in that patient subgroup. Presence of anemia (defined as a hemoglobin less than 10 g/dL) was also not associated with the primary outcome in our study. Anemia and comorbid lung disease (like COPD or asthma) in the presence of even nonsevere sepsis both represent limitations to oxygen delivery at a time when the body has a higher metabolic demand. Prior literature has shown associations with both anemia and comorbid lung disease and disease progression to septic shock [1]. It could be that the cutoff we chose for anemia was too high, especially given more recent literature showing no improvement in mortality with high blood transfusion goals compared with lower goals in sepsis [40]. AIDS was also not found to have a statistically significant association with disease progression in patients with nonsevere sepsis. AIDS is the terminal state of HIV infection, characterized by a depletion in cell-mediated immunity. The effect we expected to see in our sample is present, but modest, and disappears in multivariate analysis. This is due in part to a low absolute number of AIDS cases in our cohort, though they are relatively high as a percentage of the sample compared to similar studies [1, 37, 39].

**Table 6:** Independent predictors of sepsis progression among patients presenting with nonsevere sepsis

Predictor	Odds ratio	95 % confidence interval
Low diastolic blood pressure at ED triage (<52 mmHg)	4.59	1.57–13.4
Low initial serum albumin (<3.5 g/dL)	4.82	2.40–9.69

Any continuous predictors were dichotomized before entry into our model. For instance, diastolic blood pressure was dichotomized at 52 mmHg. This value corresponds to the diastolic blood pressure if the systolic blood pressure is 90 mmHg and the mean arterial blood pressure is 65. Clinical hypotension is defined in the literature as a systolic blood pressure less than 90 mmHg and/or a mean arterial blood pressure less than 65 mmHg. Serum bicarbonate was dichotomized at 20 mmHg, which was the lower limit of normal at the study site. Serum albumin was dichotomized at 3.5 g/dL because any value below this is considered hypoalbuminemia. Serum sodium was dichotomized at 145 mEq/L because sepsis disease progression has been associated with an initial serum sodium above that value [16]. The goal in dichotomizing continuous variables was to use predictors that would not involve complicated algorithms so they could be easily utilized by ED clinicians. This approach has been used in similar studies, based on clinically meaningful cutoffs [1, 23, 37]. Dichotomizing risk factors provides a simple tool for quick triage decisions in a busy emergency department but at the expense of losing potential predictive information and discriminatory power [41].

Our dataset had a high percentage of missing data for the components that made up our primary outcome. Our full composite outcome variable had eight components: pulmonary dysfunction, hematologic dysfunction, renal dysfunction, liver dysfunction, coagulopathy, hyperlactatemia, shock, and death, all within 96 h of ED presentation. Of these, liver dysfunction (measured by total bilirubin), coagulopathy (measured by INR and PTT), and hyperlactatemia could not be assessed on a large number of patients (25.4, 55.7, and 78.7 %, respectively) because no repeat values were sent within 96 h of ED presentation. We took two actions in our analysis to address this issue: (1) we made assumptions about missing repeated measures of organ dysfunction; (2) we performed a sensitivity analysis using outcomes in which we had <5 % missing repeated measures. If any one of these values were missing, we assumed that patients who were discharged from the hospital did not have those specific organ dysfunctions in the full composite outcome variable for disease progression. We may have introduced misclassification bias by assuming the absence of organ dysfunction from missing datapoints. After testing associations with potential predictors to our primary composite outcome of disease progression, we ran a separate analysis of a reduced composite outcome—using only respiratory failure, renal dysfunction, thrombocytopenia, or development of shock. The aim was to see if bias related to missing data may account for an absence of expected associations. We performed a sensitivity analysis with a reduced disease progression outcome variable to address this problem. Albumin <3.5 g/dL became the only independent predictor of the reduced disease progression outcome. Diastolic blood pressure <52 mmHg no longer was statistically significant in the model with reduced disease progression outcome.

Mortality within 96 h of ED presentation was included as an outcome, unlike some studies that assessed only sepsis progression [16, 23, 37, 39]. We incorporated mortality into the composite outcome, with the presumption that there may be patients in whom there is such rapid and unexpected disease progression that measurement of some of the other disease progression parameters such as organ dysfunction or hypotension may not be documented prior to patient death. There were no deaths within 96 h of ED presentation in our patient population.

Our study suffers from some important limitations. This study was based on a chart review with some missing data. Repeat laboratory tests that would indicate disease progression were not available for all patients, presumably because they were not ordered. It is probable that these data were not missing randomly. Less sick patients would be less likely to receive repeat tests than those who were sicker. Given the probable non-random distribution of missing data, imputation was not performed. In the absence of any other information, we categorized these patients as not having disease progression, thus introducing potential for misclassification bias. We conducted a sensitivity analysis by excluding these patients and found a difference in our conclusions. However, we avoided other possible limitations of chart review methodology by following strict protocol that blinded the data collection of predictors and outcomes, used standardized chart review techniques and DCIs, and systematically monitored the data collection. Another limitation is the inherent selection bias in our sample. Only patients for whom a lactate level was sent by the treating physician could be included. It is possible that clinicians in our ED only sent a lactate on sicker patients, potentially biasing our sample and limiting the generalizability of our findings. However, our sample had a lower mortality rate and similar incidence of sepsis progression compared to previously published studies with comparable objectives [1, 24].

## Conclusions

Our study demonstrated that an initial serum albumin <3.5 g/dL and an ED triage diastolic blood pressure <52 mmHg had independent, statistically significant associations with early future progression to severe sepsis or shock among patients with nonsevere sepsis. Future studies should validate our findings in a larger multicenter cohort with a low percentage of missing data. The next step would be to derive and validate a prediction tool for ED identification of nonsevere sepsis patients at increased risk of disease progression. This could lay the groundwork for further studies of interventions aimed at reducing sepsis progression in this population.
